# Supplementary material for: Inhibiting presynaptic calcium channel motility in the auditory cortex suppresses synchronized input processing
Source: Front Cell Neurosci. 2024 Apr 10;18:1369047. doi: 10.3389/fncel.2024.1369047 (PMC11041022; doi:10.3389/fncel.2024.1369047)
Supplement: Supplementary file 1 [file Image_1.pdf]

Supplemental

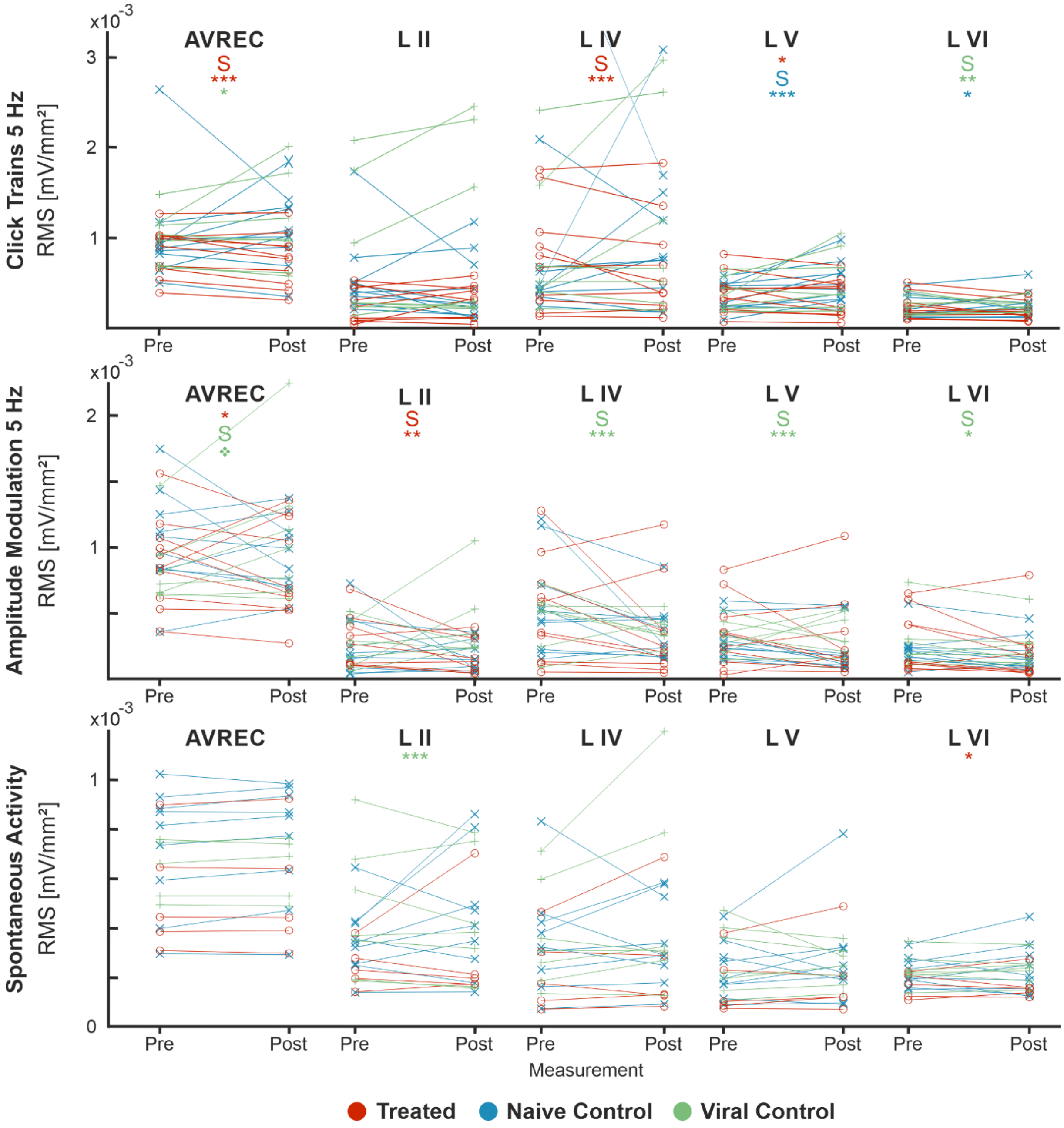

Supp Figure 1 Paired single subject data from pre- post-laser; Trial-averaged subject data paired from pre- to post-laser for CRY2olig treated (orange), naïve control (blue), and viral control (green). Data is shown for the AVREC and thalamic input layers II, IV, V, and IV RMS in response to the first 200 ms of 5 ms click trains (top), 5 ms amplitude modulated tones (middle), and during 1400 ms spontaneous activity (bottom). The RMS value of the Naïve control, layer IV, click train, pre-laser response that goes off the scale is 0.0051 mV/mm². Results for within group (in matching color) Student's *t* test and Cohen's d effect size results overlaid when significant or at least small, respectively. $p < 0.05$ *, <0.01 **, <0.001 ***, <0.000001❖, Bonferroni corrected in single-trial comparisons (n=14). Cohen's d 0.2-0.5 = small, 0.5-0.8 = medium, 0.8-1.2 = large. Within group stats are found in Tables 2 (clicks and AMs) and 7 (Spontaneous), between group stats (not visualized) in Tables 1 and 6.
